# STATE-LEVEL EDUCATION CONTEXT AS A MODERATOR OF GENETIC RISK FOR POOR COGNITIVE FUNCTION ACROSS GENDER AND COHORT

**DOI:** 10.1093/geroni/igad104.0005

**Published:** 2023-12-21

**Authors:** Eleanor Kerr, Pamela Herd, Katrina Walsemann

**Affiliations:** University of Maryland, College Park, College Park, Maryland, United States; Georgetown University, Washington, District of Columbia, United States; University of Maryland, College Park, College Park, Maryland, United States

## Abstract

APOE, specifically the e4 allele, is the strongest known genetic risk factor for late-onset dementia. Individuals with at least one copy of the e4 allele have a 3-15 times higher risk for dementia, and faster rates of cognitive decline, than those without it. Although education has been shown to moderate this relationship, the quality of U.S. education improved significantly over the first seven decades of the 20th century and saw greater access to educational opportunities among women. In this historical context, it is important to consider if genetic risk for poor cognitive function changes across successive cohorts and if it does so differentially by gender. Consequently, we use genetic and cognition data from the Health and Retirement Study (HRS) linked to state-level data on educational quality available from 1920-1974. Given well-known differences in the effect of APOE genotype on dementia risk based on global and local ancestry, we restrict our sample to non-Hispanic White men (n=5,256) and women (n=6,857) who provided at least one observation on cognitive function from 2006 – 2018. We examine the relationship across three measures of cognition – global functioning, episodic memory, and numeracy. We estimate gender and cohort stratified linear mixed models and determine if states with well-resourced education systems mitigate the genetic risk on cognitive health associated with the e4 allele and compare this relationship across cohorts for men and women. These results can help inform our understanding about how early life education can differentially shape genetic risk for cognitive decline in later life.

